# Regulation of Proteinase Activated Receptor-2 on airway epithelium

**DOI:** 10.1186/1710-1492-10-S1-A40

**Published:** 2014-03-03

**Authors:** Vivek Gandhi, Harissios Vliagoftis

**Affiliations:** 1 Pulmonary Research Group, Department of Medicine, University of Alberta, Edmonton, Alberta, T6G 2S2, Canada

## Background

The prevalence of allergic asthma has increased dramatically over the last 20 years. Environmental allergens such as house dust mites (HDM), cockroach, fungi and pollens are major asthma triggers. Recent studies indicate that the serine proteinase activity of these allergens is an important factor contributing to their ability to induce airway inflammation. Allergen serine proteinases can activate Proteinase Activated Receptor -2 (PAR-2), a G protein coupled receptor, which is upregulated on the airway epithelium of asthmatics. PAR-2 activation is pro-inflammatory in many biological systems. PAR-2 polymorphisms are associated with the development of atopy. We have shown that allergic sensitization and inflammation in mouse models of asthma is PAR-2 dependent. We have proposed that PAR-2 on the airway epithelium is a sensor for environmental allergens and leads to allergic inflammation. However, the regulation of PAR-2 expression on airway epithelium is poorly studied. As asthmatic airways are under various types of cellular stress, we hypothesized that cellular stress regulates PAR-2 on airway epithelium.

## Methods

To study the effect of cellular stress on PAR-2 expression, Normal Human Bronchial Epithelial (NHBE) cells were exposed to various stressors such as inflammatory mediators, hypoxia, growth factor deprivation, ROS (Reactive Oxygen Species) and RNS (Reactive Nitrogen Species) for various time periods and PAR-2 mRNA levels were studied by real time PCR. PAR-2 function in stressed cells was assessed by measuring IL-8 release following activation with PAR-2 specific activating peptide (PAR-2 AP).

## Results

Growth factor deprivation significantly upregulated PAR-2 mRNA (2.25 +/- 0.2 fold), while all the other studied cellular stress stimuli did not modulate PAR-2 expression on airway epithelial cells. Growth factor deprived cells showed significantly upregulated PAR-2 mediated IL-8 release (2.1 +/- 0.2 fold) compared to cells grown with growth factors. Addition of epinephrine, a growth medium supplement used for airway epithelial cells, prevented the effects of growth factors deprivation on PAR-2 expression.

## Conclusion

Cellular stress could be the driving force for increased PAR-2 expression in asthmatic airways. Further activation of this upregulated PAR-2 can perpetuate inflammation by releasing higher levels of inflammatory mediators. Epinephrine, an adrenergic agonist, neutralizes stress effect on PAR-2 expression. Understanding the mechanisms of these effects could lead to the development of more specific treatments for preventing PAR-2 mediated airway inflammation.

